# When Mammary-Like Anogenital Glands Go Rogue: Vulvar Phyllodes Tumor With Incorporated Hidradenoma Papilliferum

**DOI:** 10.7759/cureus.116700

**Published:** 2026-09-22

**Authors:** Mariam N Albrahim, Rajan Arora, Ramani Samudrala

**Affiliations:** 1 Pathology and Laboratory Medicine, Farwaniya Hospital, Farwaniya, KWT; 2 Obstetrics and Gynecology, Farwaniya Hospital, Farwaniya, KWT

**Keywords:** anogenital mammary-like glands, hidradenoma papilliferum, phyllodes tumor, vulvar neoplasm, vulvar phyllodes tumor

## Abstract

Vulvar phyllodes tumor is a rare fibroepithelial neoplasm with morphologic features resembling mammary phyllodes tumor. Hidradenoma papilliferum is a benign anogenital neoplasm associated with mammary-like anogenital glands. We report a 55-year-old female patient with a three-year history of an asymptomatic right labial mass clinically diagnosed as a sebaceous cyst. Histopathologic examination revealed a benign phyllodes tumor with an incorporated glandular proliferation consistent with hidradenoma papilliferum. Immunohistochemistry demonstrated a p63-positive myoepithelial layer. To the best of our knowledge, this association has not been previously reported. This case expands the recognized morphologic spectrum of mammary-like gland-associated vulvar neoplasia and highlights the importance of recognizing both patterns within a single lesion.

## Introduction

Vulvar phyllodes tumor is a rare fibroepithelial neoplasm characterized by a biphasic epithelial and stromal proliferation that closely parallels the morphology of mammary phyllodes tumor [1,2]. Histologically, it typically exhibits an intracanalicular growth pattern with characteristic leaf-like architecture [2]. Despite these morphologic similarities, the histogenesis of this tumor remains controversial. Earlier theories proposed that these lesions arise from ectopic mammary tissue located along the embryonic milk line, whereas more recent evidence favors an origin from mammary-like glands of the anogenital region [2,3].

Hidradenoma papilliferum is one of the most common benign neoplasms of the female anogenital region and is also believed to arise from mammary-like anogenital glands [4]. Histologically, it is typically composed of well-circumscribed cystic and papillary structures lined by epithelial and myoepithelial cells, often with evidence of apocrine differentiation [4]. Clinically, these lesions may resemble more common benign vulvar masses; histopathologic evaluation is essential for accurate classification. The occurrence of a vulvar phyllodes tumor containing an incorporated hidradenoma papilliferum component is unusual and presents an additional diagnostic consideration. Its occurrence within a single tumor broadens the recognized histologic spectrum of neoplasms associated with mammary-like glands of the anogenital region.

## Case presentation

A 55-year-old female patient presented with a three-year history of an asymptomatic right labial mass. Physical examination revealed a firm, mobile, well-circumscribed lesion measuring approximately 3 × 2 cm. The patient reported no interval change in size or associated tenderness. The clinical impression was a sebaceous cyst, and the lesion was subsequently excised without complication. Histopathologic examination demonstrated a well-circumscribed, non-encapsulated biphasic fibroepithelial neoplasm characterized by prominent intracanalicular growth (Figure 1).

**Figure 1:**
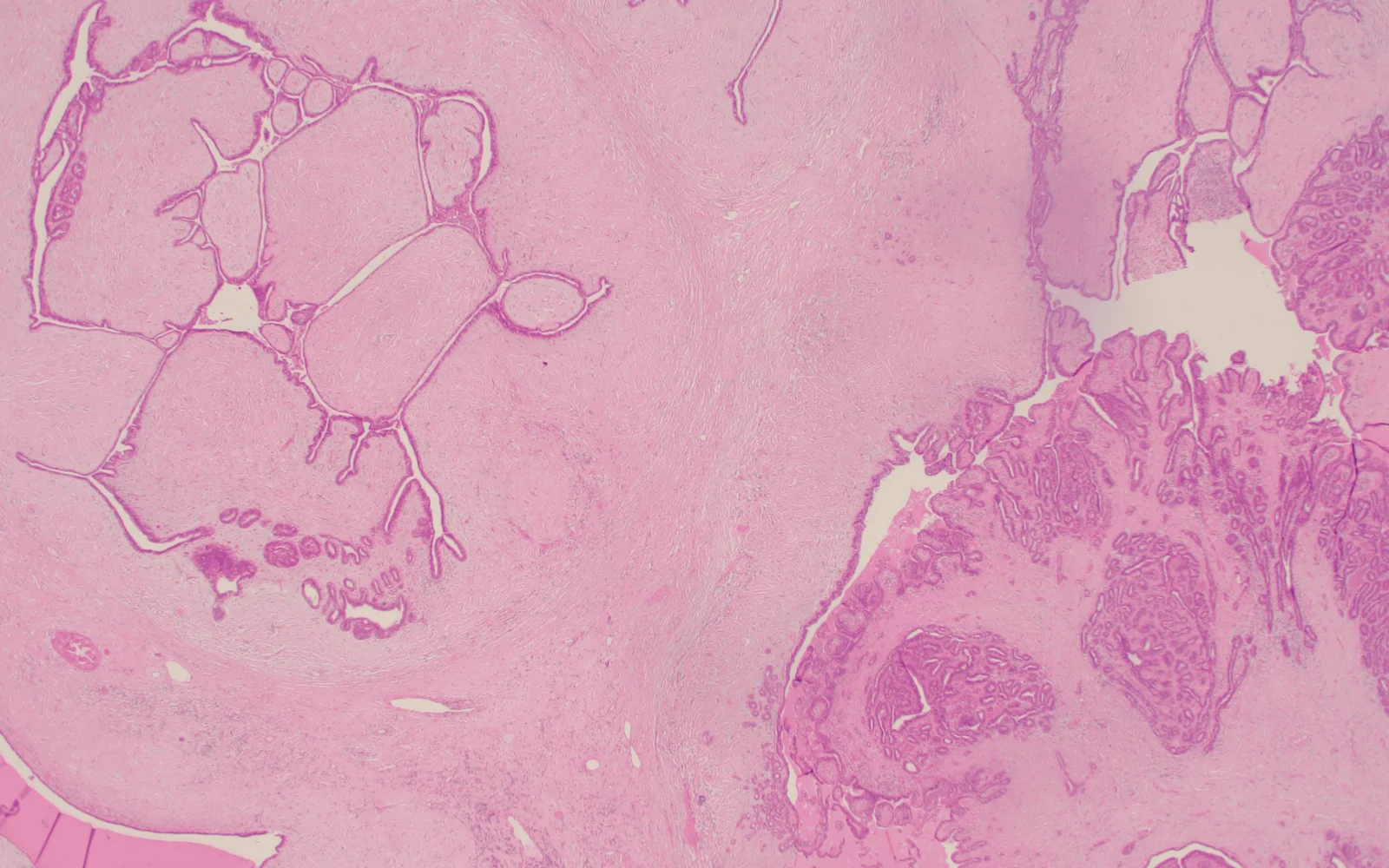
Histopathology showing the biphasic phyllodes tumor component on the left and the incorporated hidradenoma papilliferum component on the right (H&E, x20) H&E: hematoxylin and eosin

Cleft-like spaces lined by bland epithelium were surrounded by mildly cellular stroma, producing characteristic leaf-like architecture. The stroma showed focal sclerosis and chronic inflammation without significant nuclear atypia or stromal overgrowth. Mitotic activity was low, with up to 1 mitosis per high-power field. No stromal condensation was noted around the epithelial structures, and the resection margin was free of the lesion (Figure 2). These findings supported classification as a benign phyllodes tumor.

**Figure 2:**
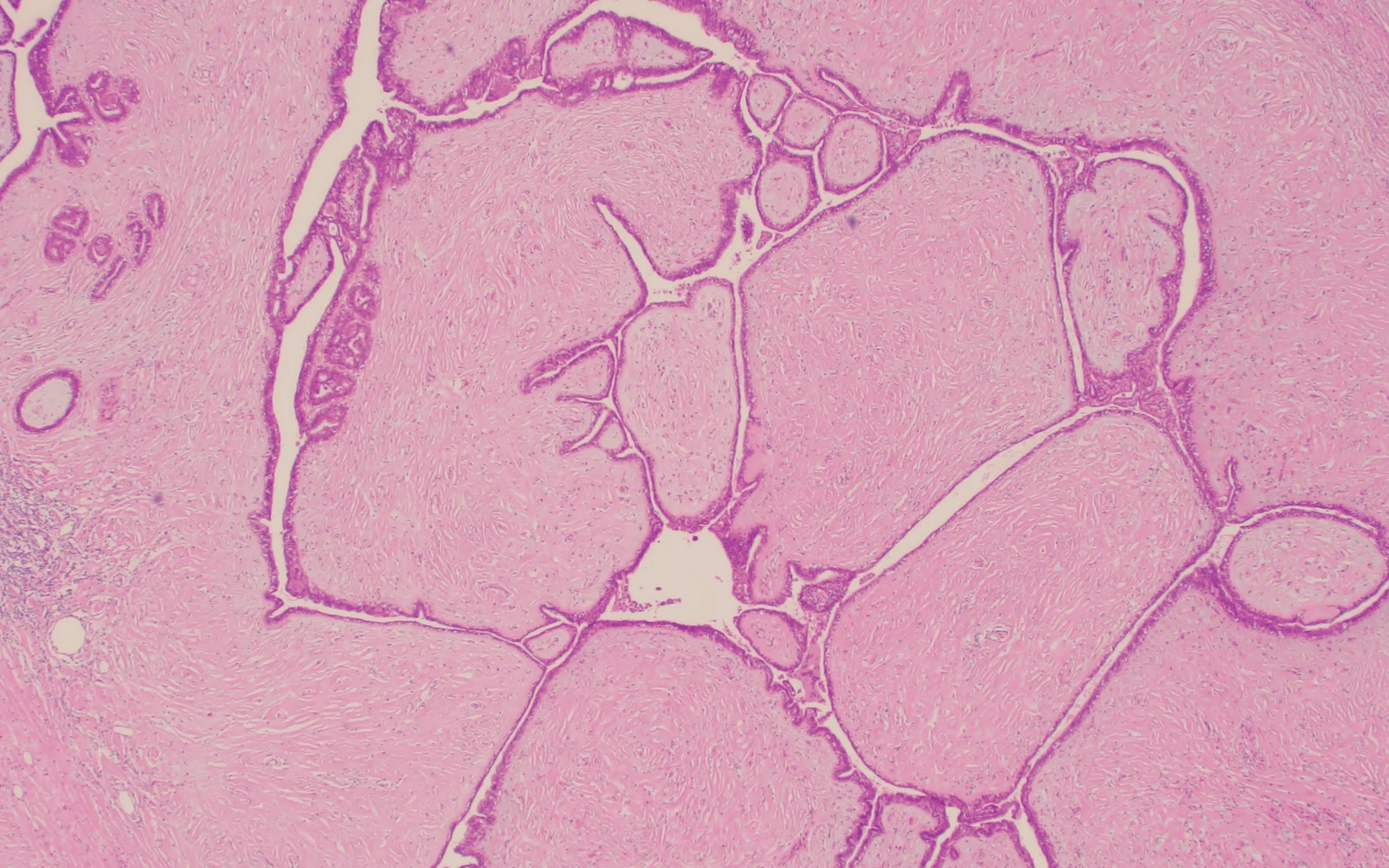
Phyllodes tumor showing leaf-like architecture (H&E, x40) H&E: hematoxylin and eosin

In addition, an incorporated glandular proliferation consistent with hidradenoma papilliferum was identified, composed of bilayered glands lined by inner epithelial and outer myoepithelial cells, with focal papillary architecture and squamous differentiation (Figure 3). Immunohistochemical staining with p63 highlighted the preserved myoepithelial layer, further supporting the diagnosis (Figure 4).

**Figure 3:**
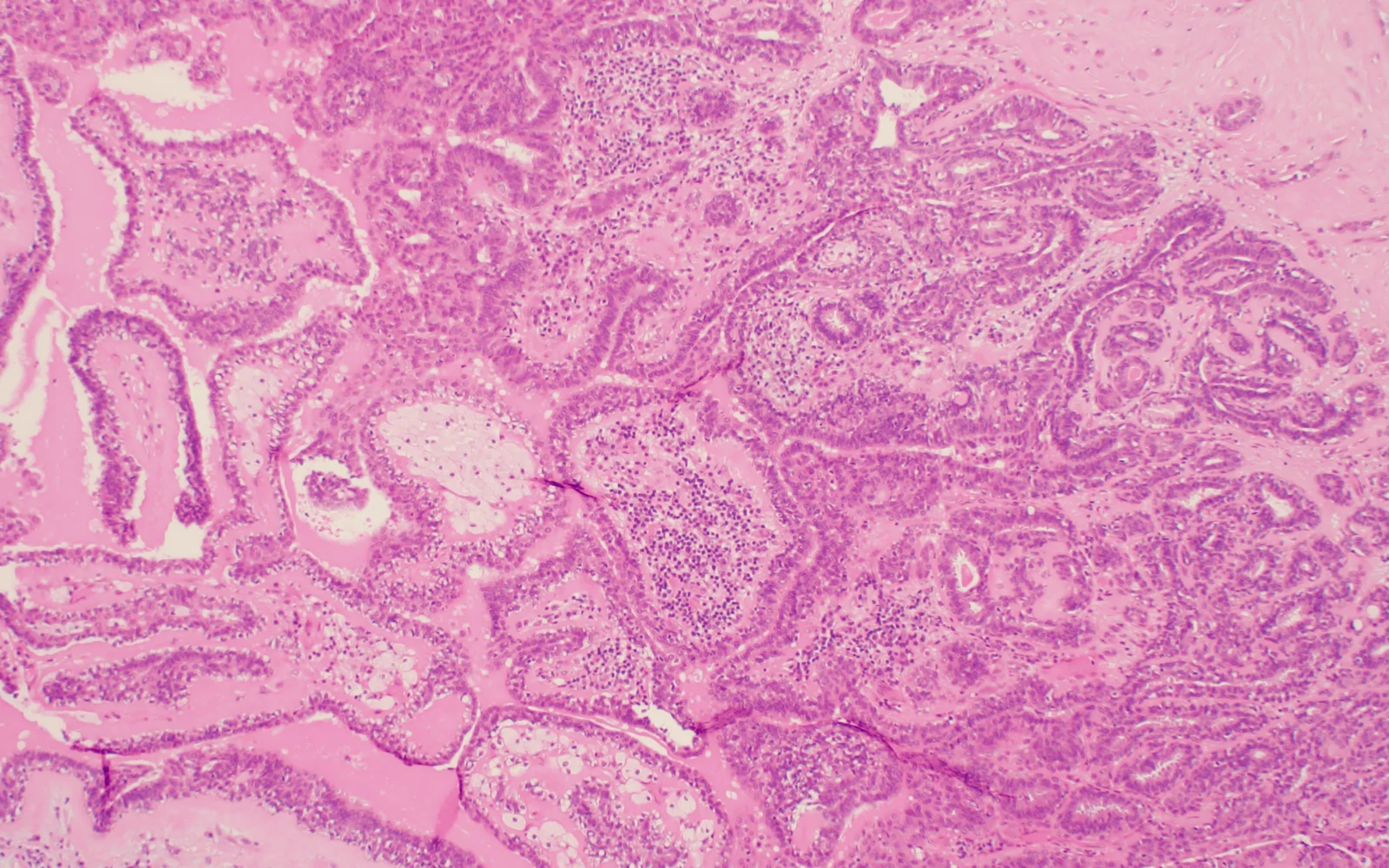
Hidradenoma papilliferum demonstrating characteristic papillary architecture (H&E; x100) H&E: hematoxylin and eosin

**Figure 4:**
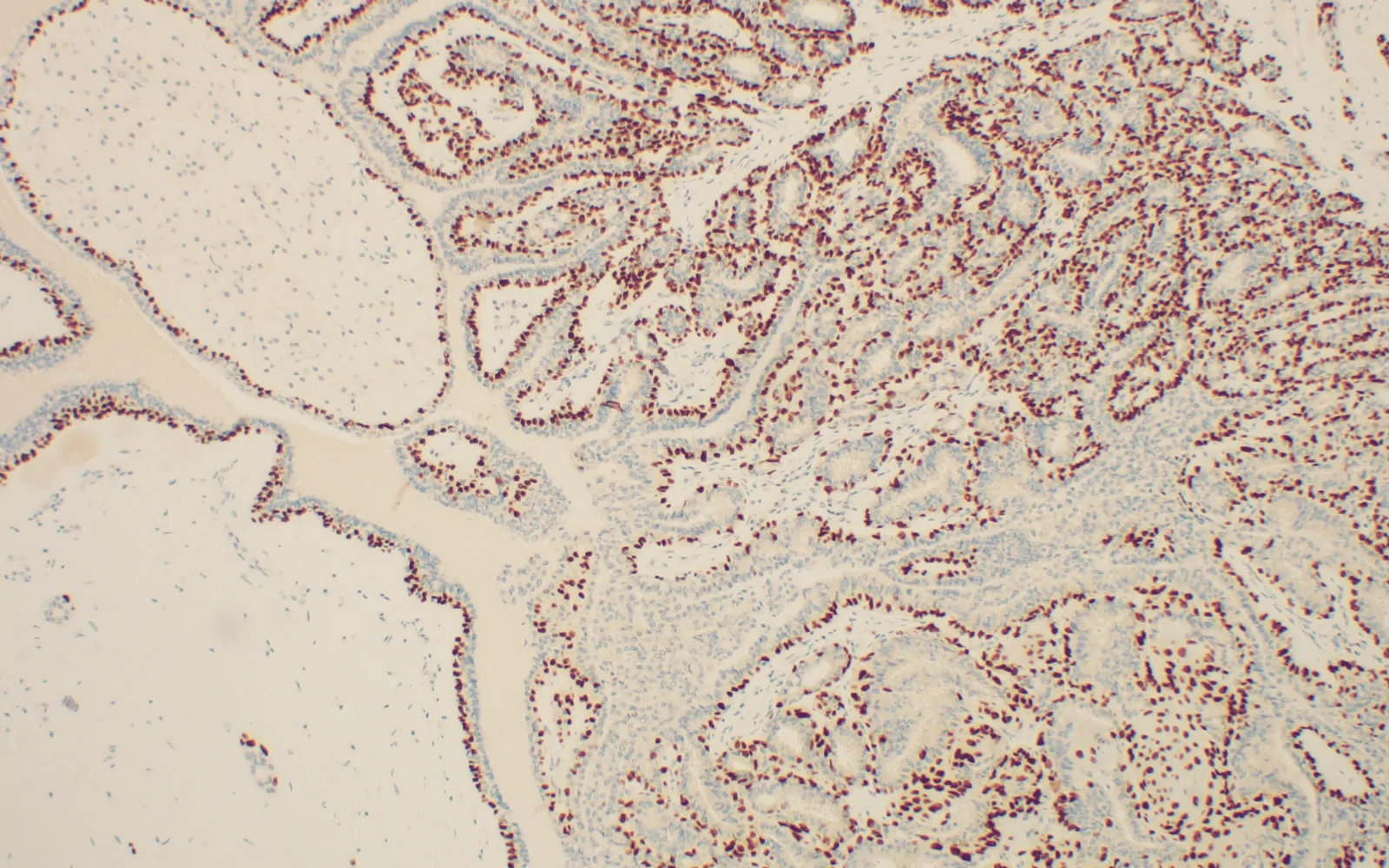
A p63 immunohistochemical stain highlighting the outer myoepithelial layer

Overall, the histopathologic and immunohistochemical findings supported the diagnosis of a benign phyllodes tumor with incorporated hidradenoma papilliferum. No additional treatment was required. At seven months of follow-up, the patient remained well with no evidence of local recurrence.

## Discussion

Vulvar phyllodes tumor is an uncommon fibroepithelial neoplasm with morphologic features analogous to those of phyllodes tumor of the breast [1,2]. The characteristic biphasic epithelial and stromal proliferation with leaf-like architecture is recognizable on histopathologic examination; however, its occurrence in the vulva has prompted considerable debate regarding histogenesis [2]. Historically, breast-type vulvar lesions were thought to arise from ectopic mammary tissue derived from remnants of the embryonic mammary ridges. More recently, the recognition of mammary-like glands as normal constituents of the anogenital region has provided an alternative and increasingly accepted explanation for the development of these lesions [2,3,5]. The present case is unusual in demonstrating hidradenoma papilliferum incorporated within a benign vulvar phyllodes tumor.

Mammary-like glands are normal constituents of the anogenital region, most commonly located within the interlabial sulcus [5]. Histologically, they show features resembling mammary tissue, including epithelial and myoepithelial components and occasional apocrine differentiation [4,5]. A broad spectrum of breast-type lesions has been associated with these glands, including fibroadenoma, hidradenoma papilliferum, intraductal papilloma, sclerosing adenosis, phyllodes tumor, and adenocarcinoma [5,6]. Their recognition challenges the earlier concept that breast-type vulvar lesions arise from ectopic mammary tissue along the embryonic milk line and instead supports the interpretation of these glands as native structures of the anogenital region [3,5]. 

Clinically, both vulvar phyllodes tumor and hidradenoma papilliferum may present as slow-growing, painless vulvar or labial masses that resemble more common benign lesions [1,4]. This clinical overlap can make preoperative diagnosis difficult, particularly when both morphologic patterns are present within the same mass. In our patient, the lesion had been present for three years without appreciable growth or tenderness and was clinically interpreted as a sebaceous cyst. Histopathologic examination confirmed a benign phyllodes tumor with an incorporated glandular proliferation showing features of hidradenoma papilliferum. Immunohistochemistry further supported the diagnosis by highlighting myoepithelial differentiation within the glandular component.

The differential diagnosis includes several biphasic, stromal, and adnexal vulvar lesions with overlapping morphologic features, particularly fibroadenoma, aggressive angiomyxoma, cellular angiofibroma, and superficial myofibroblastoma [2,7]. Their principal distinguishing features are summarized in Table 1.

**Table 1:** Differential diagnosis of vulvar phyllodes tumor and key distinguishing features [2,7]

Category	Differential diagnosis	Key distinguishing features
Fibroepithelial lesions	Fibroadenoma	Biphasic fibroepithelial lesion with generally lower stromal cellularity and less pronounced intracanalicular growth; prominent leaf-like architecture favors phyllodes tumor.
Phyllodes tumor grading	Borderline or malignant phyllodes tumor	Increased stromal cellularity, cytologic atypia, mitotic activity, stromal overgrowth, and infiltrative margins. Malignant tumors may additionally show marked stromal pleomorphism and heterologous sarcomatous differentiation.
Stromal tumors of the lower female genital tract	Deep (aggressive) angiomyxoma	Deep, poorly circumscribed, and infiltrative neoplasm composed of bland spindle or stellate cells in abundant myxoid stroma with prominent vessels; lacks a biphasic epithelial-stromal architecture.
	Cellular angiofibroma	Well-circumscribed cellular spindle-cell neoplasm with numerous thick-walled or hyalinized vessels; lacks epithelial-lined clefts and leaf-like architecture.
	Superficial myofibroblastoma	Superficial, well-circumscribed stromal neoplasm with a characteristic subepithelial grenz zone and numerous thin-walled vessels; lacks biphasic fibroepithelial architecture.
Müllerian lesions	Adenosarcoma	Biphasic neoplasm composed of benign or mildly atypical glands accompanied by malignant stromal proliferation, typically showing periglandular stromal condensation and often leaf-like architecture; the malignant stromal features distinguish it from benign phyllodes tumor.

## Conclusions

To the best of our knowledge, based on the available English-language literature, this association has not been previously reported. The incorporation of hidradenoma papilliferum within a vulvar phyllodes tumor broadens the recognized morphologic spectrum of mammary-like gland-associated vulvar neoplasia and highlights the diagnostic importance of recognizing this unusual combined pattern. Careful histopathologic evaluation is essential in clinically nonspecific vulvar masses, particularly when more than one morphologic pattern is present, as accurate recognition of each component is necessary for appropriate classification and diagnosis.
